# Simple rules for construction of a geometric nest structure by pufferfish

**DOI:** 10.1038/s41598-018-30857-0

**Published:** 2018-08-17

**Authors:** Ryo Mizuuchi, Hiroshi Kawase, Hirofumi Shin, Daisuke Iwai, Shigeru Kondo

**Affiliations:** 1 Department of Bioinformatics Engineering, Graduate School of Information Science and Technology, Osaka University, 1-5 Yamadaoka, Suita, Osaka, 565-0871 Japan; 2 Coastal Branch of Natural History Museum and Institute, Chiba, 123 Yoshio, Katsuura, Chiba, 299-5242 Japan; 3 Graduate School of Engineering Science, Osaka University, 1-3 Machikaneyama, Toyonaka, Osaka, 560-8531 Japan; 4 Graduate School of Frontier Biosciences, Osaka University University, 1-5 Yamadaoka, Suita, Osaka, 565-0871 Japan; 5 Present Address: Department of Chemistry, Portland State University, PO Box 751 Portland, OR 97207 USA

## Abstract

A small (~10 cm) male pufferfish (*Torquigener albomaculosus*) builds a large (~2 m) sandy nest structure, resembling a mysterious crop circle, to attract females. The circle consists of radially arranged deep ditches in the outer ring region, and maze-like shallow ditches in the central region. The configuration is geometrical. Here, we examined the process of the outer ring construction, and extracted the ‘rules’ followed by the pufferfish. During construction, the pufferfish repeatedly excavates ditches from the outside in. Generally, excavation starts at lower positions, and occurs in straight lines. The entry position, the length, and the direction of each ditch were recorded. A simulation program based on these data successfully reproduced the circle pattern, suggesting that the complex circle structure can be created by the repetition of simple actions by the pufferfish.

## Introduction

Many animals build diverse and complex forms of nests, which protect their eggs and offspring from predators, and can also act as a signal to attract mates^1,2^. The structures of some of the nests, such as those hosting colonies of ants or termites, and honeycombed structures of wasps and bees, are well organized and much larger than the body size of the builders. How they are able to construct such a geometrical structure is an interesting ecological question to be addressed. Previous studies, using computer simulations, suggested that the behaviors of social insects do not depend on the whole nest structure during construction, and are rather simply determined by local physical or chemical conditions, and the structural patterns gradually appear through the repetition of local behaviors^3–12^. Other studies indicated that orb-web spiders could also build their ordered webs by repeating simple actions without recognizing the whole structure^13,14^.

**Figure 1:**
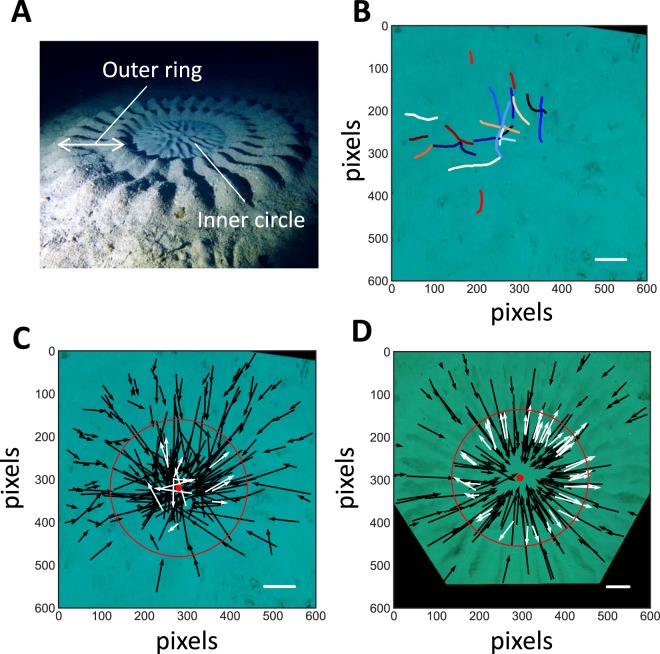
Outer-ring construction by a male pufferfish. (**A**) Photo image of a pufferfish nest (final-stage). (**B**) The trajectories of first 20 (of 229) consecutive excavations by a male pufferfish in the early stage on June 8, 2016, obtained by tracing the location of the dorsal fin in every 0.5 seconds. (**C**,**D**) The simplified trajectories of the same pufferfish. 229 consecutive excavations in the early stage on June 8 (**C**) or 191 consecutive excavations in the middle stage on June 12, 2016 (**D**). The black and white vectors represent inward and outward excavations, respectively. The red points and circles represent the centers of the nests and the inner edges of appearing outer ring structures, respectively. All the trajectories were obtained from rectified video images. The background images are the rectified photos at the timing of initial excavations. The white lines displayed in the lower right corners show the body length of the pufferfish. The black space region was out of the video.

In 1990s, on the seabed around Amami Oshima Island, southwest Japan, native divers discovered a mysterious crop circle-like structure. The circle was approximately 2 meters in diameter, and consisted of two distinct shapes, radially arranged as 25–30 deep ditches in the outer-ring region, and shallow maze-like ditches in the central region (Fig. 1A). Recently, Kawase *et al*. found that the large circle is a nest structure built by a small male pufferfish, *Torquigener albomaculosus*, approximately 10 cm in length, to attract female mates^15^. The nest structure is much more geometrically ordered than any known nests built by other fish^1,2,16–19^.

An intriguing question about this mysterious circle is how the small pufferfish could construct the large and regular structure accurately. To initiate the construction, a pufferfish makes a mark by pushing its belly on the sandy bottom as the central region of a nest (Supplementary Fig. S1A)^20^. Next, it repeatedly excavates the sand with its fins and body to leave marks hundreds or thousands of times, during which the radial ditches in the outer ring region gradually appear^15,20^. Thereafter, it excavates the inner region to form a maze-like structure. According to the video recordings shown in the previous reports^15,20^, the pufferfish mainly stayed near the sea bottom during the construction, suggesting the possibility that the structural patterns would spontaneously emerge by the pufferfish repeating some simple behavior triggered by a signal, which is not directly related to the whole structure of the nest.

Here, we statistically analyzed pufferfish behavior to obtain the general rules for construction of the radial deep ditches formation in the outer-ring region. Kawase *et al*. (2017) reported that the process of the nest construction is divided into three stages^20^. The early stage is from the beginning until the basic circular shape emerges. In the middle stage, the fish completes the radial ditches in the outer circle. In the final stage, the fish constructs the maze pattern in the inner circle. (For detail, see Kawase *et al*., 2017.) In this study, we analyzed the excavation behavior of a male pufferfish during the early and the middle stages. We then determined a simple rule governing the excavation behaviors, and a computer simulation confirmed that the repetition of simple actions generates highly ordered structural patterns, similar to the geometric ring structure of the pufferfish nest.

## Results

### Statistical analysis of excavation behavior

#### Rectifying the video image to view the image from directly above the nest

As the pufferfish stays at the bottom of the sea during nest construction, videos would ideally be recorded from directly above the nest. However, because of technical restrictions, all the videos were recorded from diagonally upwards of the nest. Therefore, the angle and the length of each excavation could not be taken directly from the 2D vision of the video. To overcome this problem, before analyzing the fish behavior, we converted the video image to that from the angle directly above the nest. Details of this calculation are explained in the materials and methods.

#### Each excavation can be represented by a vector

During nest construction, the pufferfish repeatedly excavates ditches thousands of times. Video recordings of this process suggest that each excavation goes along a straight line. To confirm this, we traced the dorsal fin in each frame of the video, and plotted the trajectory. Fig. 1B shows the trajectories of 20 consecutive excavations made by the pufferfish during the early stage of the nest formation, when the radial ditches were not yet clear. Each trajectory appears to be in nearly a straight line. To quantitate the straightness of the trajectories, the average straightness index^21^ was calculated to be 0.97 (Supplementary Table S1), which confirms that the trajectories are almost straight. Even in cases when the pufferfish encountered a premature ditch running across the excavating line, the pufferfish maintained its direction by destroying the pre-formed immature ditches (Supplementary Movie S1). From these observations, we concluded that each excavation behavior can be simplified as a straight arrow (vector) for later analysis. Figs. 1C,D show the consecutive trajectories of early (219 excavations) and middle (191 excavations) stages of the nest construction (The total number of excavation through the nest construction is about 10000. This number was approximated from (frequency) × (total time)^20^).

#### Parametric representation of each excavation for statistical analysis

**Figure 2:**
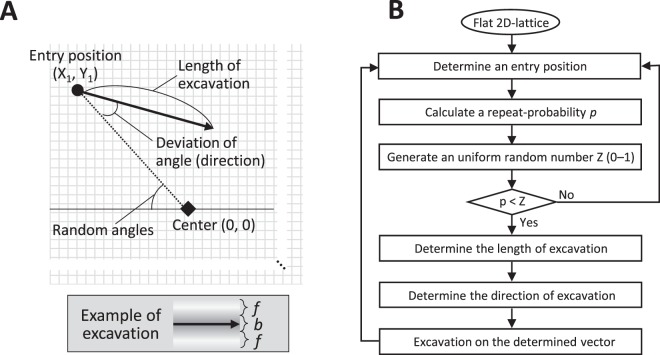
Simulation model for nest construction behavior of pufferfish. (**A**) The schematic representation of the simulation. Briefly, on a two-dimensional square lattice, using the measured parameters (Table 1), the trajectory of excavation (vector) was obtained by determining an entry position (filled circle), a length of excavation, and the direction of excavation. The angles of the entry positions to the center were randomly chosen. Excavation occurs on the vector. The example of excavation below shows the change of heights by the excavation, defined in dependence on the assumed body width of pufferfish (b) and the factor of sand diffusion (f). Darker color represents lower sites. (**B**) The algorithm of the developed simulation. The details were described in the main text.

Each excavation is defined by the following parameters: the “entry position”, “direction of excavation” and “length of excavation”, as shown in Fig. 2A. Histograms for the measured values and obtained parameters are shown in Supplementary Fig. S2 and Table 1, respectively. They are values normalized by the body length of the pufferfish. In order to measure the value of each parameter, the center position of the nest is required. At the beginning of the nest construction, the pufferfish roughly marked the center region by pushing its belly on the sandy bottom (Supplementary Fig. S1A)^20^. The landmark could consist of the shape of the sand’s surface that is visually identified by the fish, or could consist of some chemical cue, such as a pheromone. In any case, it is reasonable to assume that the pufferfish knows the location of the center position at least roughly when it excavates. We hypothesized that the center landmark position for the pufferfish is identical to the point where most of the trajectory vectors meet.

**Table 1:** Parameters of excavation by a male pufferfish

	Jun. 8^th^ (Early stage)	Jun. 12^th^ (Middle stage)
Number of inward excavations from the ring region	125	81
Normalized length between the entry position and the center	3.4 ± 0.77	4.8 ± 1.0
Deviation of the direction of the excavation (radian)	0.28	0.094
Normalized length of the excavation	1.1 ± 0.69	1.8 ± 0.96

“Normalized length between entry position and the center” and “Normalized length of excavation” are shown as mean ± standard deviation. The parameters were normalized by the body length of the pufferfish. Raw data used for the analysis were shown in (Supplementary Fig. S2). “Deviation the direction of excavation (radian)” was obtained as the standard deviation of “Angle of the direction of the excavation to the center” in the figure.

#### Two distinct areas: inner circle and outer ring

The completed nest is composed of two distinct areas, the inner circle region, which has a maze pattern, and the outer ring region, which has radial ditches. During the early and middle stages of nest construction, only the radial ditches in the outer ring are made. However, as shown in Fig. 1C,D, the pufferfish excavated not only in the outer ring region but also excavated extensively in the inner circle region. This is curious because the excavation in the inner circle during this stage does not contribute to the completed structure. The excavation in the outer ring always moved inwards, towards the center position. However, the excavation in the inner circle contains many outward movements (white vector, Fig. 1C,D). It is clear that in the middle stage, the exit position of the outward excavation is concentrated at the boundary of the inner circle and the outer ring region. Based on these observations, we assumed that the aim of the inner circle excavation is not to make the ditch but possibly to determine the size of the inner circle region (discussed further below). In subsequent simulation analyses, we omitted the inner circle excavation because it does not affect radial ditch pattern in the outer ring.

#### Distance between the center and the entry position

The average distance between the center and the entry position was 3.4 ± 0.77 and 4.8 ± 1.0 for early and middle stages of the nest formation, respectively (Table 1). As shown in Supplementary Fig. S2A, the distance was not fixed to the outer boundary of the nest, but was distributed almost randomly in the outer ring area.

#### Angle of the entry position to the center

In the early stage of nest construction, the data (Supplementary Fig. S2B) suggests that the pufferfish has a tendency to construct one region more extensively than other regions of the nest (Rayleigh test, p = 2.8 × 10^−9^). However, in the middle stage, when the ditches were already being constructed, the excavations were evenly distributed around the rough angle of the entry position (Rayleigh test, p = 0.29). More important is the fine angle correlated to the growing ditches. In the middle stage of nest construction, when the ditches were already clear, all the excavation began in the valley region (Supplementary Fig. S3). On the other hand, in the early stage, when the ditches were not yet clear, this tendency was not observed (Fig. 1C). From these observations, we can reasonably assume that the pufferfish recognizes the local topography when selecting the entry position. This preference of the fish would amplify local undulation of the sand surface and lead to the emergence of the final nest pattern. Therefore, in the simulation, we postulated that the pufferfish is reluctant to select a higher place as the entry position.

#### Direction of excavation vectors

When the pufferfish excavated ditches of the outer ring, they always ran inward, suggesting that pufferfish are able to recognize the center position of the nest. The direction to the center position was less accurate in the early stage of nest construction, but it became more accurate in the middle stage. From the obtained data (Supplementary Fig. S2C), the deviation of angle (radian) is ±0.28 in the early stage, and ±0.094 in the middle stage (0 is the angle to the exact center position) (Table 1).

#### Length of excavation

The length of each excavation was measured by the distance between the entry position and the exit position (Supplementary Fig. S2D). The length of excavation was 1.1 ± 0.69 and 1.8 ± 0.96 in the early and middle stages, respectively (Table 1).

### Development of the simulation model for the nest construction

This section explains how the ditch formation was calculated in the simulation (Fig. 2). The sand bottom of the sea is represented by a two dimensional lattice (x, y), and the height of each position is h(x, y). At the beginning of the nest construction, we set h at 0 for all positions. The center position that is marked by the pufferfish is (0, 0). On this lattice, a model pufferfish repeats excavations.

The entry position for each excavation is

$$({{\rm{X}}}_{{\rm{n}}},{{\rm{Y}}}_{{\rm{n}}})=(r\,\cos \,\theta ,\,r\,\sin \,\theta )$$

where *θ* is randomly sampled from a uniform distribution between 0 and 2π, and *r* is a random value sampled from the Gaussian distribution with the obtained average and the standard deviation (Table 1). To imitate the tendency of pufferfish to dislike higher places as the entry position, the selected position was discarded with the probability of *p*, and new values were resampled for the next entry position.

$$p=\{\begin{array}{cc}0.5+0.5h, & h\le H\\ \,1,\, & h > H\end{array}$$

Here, H is the highest limit that the pufferfish can start excavation. The direction and the length of each excavation was calculated using the average and the normal deviation obtained by the statistical analysis (Table 1).

The excavation area was set as the rectangle centered by the excavation vector. To simplify the movement of the sand, we assumed that the sand raised by the pufferfish would pile up at the side area to form a normal distribution curve, depending on the body width of pufferfish *b* and the factor of diffusion distance *f* (Fig. 2A and Supplementary Fig. S4). When pufferfish excavate a ditch that is already very deep, the raised sand should fall on the slope of the ditch and slide down to the original place. This physical effect should determine the limit of the depth of the ditch. To reproduce this effect with a simple calculation, we assumed that the amount of excavated sand at each time step decreased depending on the depth of the ditch where the pufferfish was excavating (see materials and method).

### Simulation result

**Figure 3:**
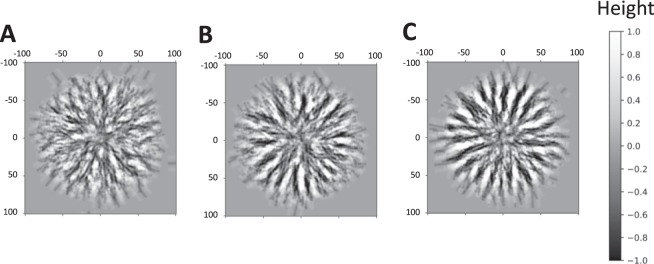
Emergence of radial ditches in the outer-ring region. (**A**–**C**) Snapshots of simulations using the early-stage parameters with no preference of entry position (A) p was set as 0 for all excavations), mild preference (**B**) H = 0.15), and strong preference (C, H = 0). p is the possibility that the fish discards the selected entry position. H is the highest limit that the pufferfish can start excavation. One scale of the simulation was estimated as 0.80 cm from the previous study^20^. Excavation was repeated 2000 times for each simulation. b = 6, f = 10.

First, we performed the simulation to find the appropriate value of the height preference at the entry position. In this test, we used the early-stage parameters, which had very variable entry positions and directions. When no preference of entry position was set, no distinct radial pattern emerged (Fig. 3A). However, by adding the condition that the pufferfish mildly avoid higher places as the entry position (H = 0.15), some short radial ditches emerged (Fig. 3B) With more clear avoidance of higher places as the entry position (H = 0), the simulation generated slightly skewed but cleary radial ditches, even when the direction of excavation varied (Fig. 3C, and Supplementary Movie S2). From this result, we set as H = 0 in the following simulation.

**Figure 4:**
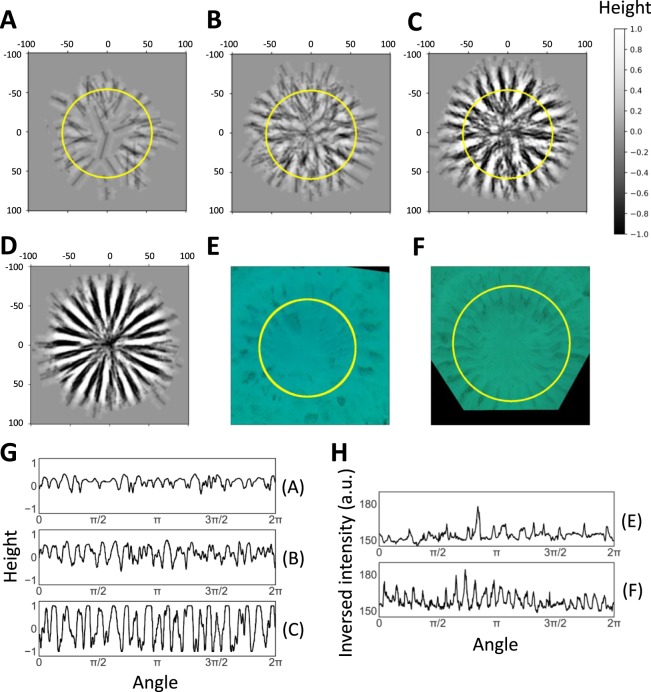
Formation of radial ditches over time. (**A–C**) Snapshots of a simulation with the early-stage parameters (H = 0, b = 6, f = 10) when excavation was repeated (**A**) 100, (**B**) 400, and (**C**) 2000 times. Yellow circles represent areas where height was analyzed. (**D**) A snapshot of a simulation using the middle-stage parameters (H = 0, b = 6, f = 10). One scale of simulation was estimated as 1.1 cm. Excavation was repeated 2000 times. (**E**,**F**) Rectified photos of (**E**) the early and (**F**) the middle stage at the end of the analysis in Fig. 1C,D. (**G**) Height over the middle part in the outer ring region through the repeat of excavation in the simulation (**A**–**C**). (**H**) Inversed image intensity (high values represent darker sites) of the middle part in the early- and the middle-stage outer ring region from the photo images (**E**,**F**). It should be noted that this intensity does not correctly represent relative heights of the nests (they are biased by many factors such as the location of the camera and the direction of sunlight).

Next, we observed the time course of the simulated nest formation. Figs. 4A–C show the results at 100, 400 and 2000 excavations, respectively. The depth of the groove in the middle part of the ring (indicated by the yellow line) is represented by the 1D graph (Fig. 4G) and visualizes the completeness of the nest. As shown in Fig. 4A, the directionality of the trajectories varied significantly in the beginning of the nest formation. However, simply repeating the excavation with the identical parameter, the nest gradually developed to a more accurate form. After the early radial ditches were formed, the pufferfish uses the ditches to determine the direction of excavation, which makes the deviation of angle smaller, as observed in the middle stage excavation (Table 1). Using the parameters of the middle stage, the simulation generated a similar but less skewed radial pattern (Fig. 4D, and Supplementary Movie S3). In Fig. 4H, we show the estimated depth graph of the real nests for comparison. Although these data are rough estimation calculated from the shadow of the photo images (Figs. 4E,F), the growth of the nest looks similar to the simulated data.

**Figure 5:**
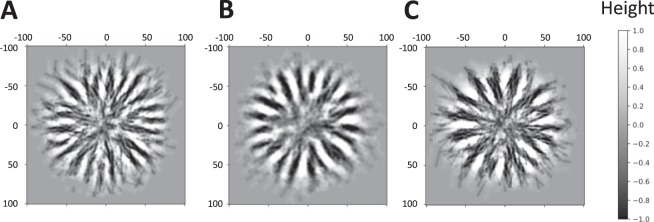
Pattern formation with different pufferfish characteristics. The simulations were performed using the early-stage parameters with different body width of pufferfish and the factor of sand diffusion. b, f = (**A**) 6, 10, (**B**) 10, 10, (**C**) 6, 14. Excavation was repeated 2000 times for each simulation.

We further investigated how the variation of pufferfish influences the radial patterns in the simulation. We found that an increased body width of pufferfish (*b*, 167%) produced a smaller number of ditches (Figs. 5A,B), and a similar result was obtained with an increased factor of sand diffusion (*f*, 140%) (Fig. 5C). These results suggest that the physical traits of pufferfish may influence the geometric pattern of nest structures.

## Discussion

In this report, we statistically analyzed the behavior of pufferfish constructing the radial ditch pattern of their mysterious circular nests, and identified several characteristics of the excavation that should lead to a better understanding of the logic underlying nest geometry. The specific features of the excavation we deduced from the video recordings were as follows:

1. Each excavation is on a short straight line.
2. There are two distinct rules that govern the excavation of outer ring region and the inner circle region.
3. Excavations in the outer ring are always toward the center of the nest, although the angle of this has significant error in the early stage. Later in the middle stage, it becomes more accurate.
4. The entry position in the outer ring region is almost random in the early stage, but in the middle stage, excavation starts only from the ditch region.
5. The length of each excavation is not determined.

Using these characteristics, we constructed a simple simulation program, which successfully reproduced the radial ditch formation in the early stage of nest formation. This shows that the characteristics listed above are useful to understanding the logic of nest formation by the pufferfish.

Additional information we could obtain from the simulation was the relationship between the physical status of the fish and the generated pattern. As shown in Fig. 5, at least in our simulation, larger fish or stronger fish that pushes the sand farther generated the ditch pattern with wider spacing. This result suggested that the female fish may know the body size of the male fish from the nest pattern. In some species, nest designs are regarded as extended phenotypic signals, which influence mate choice by attracting partners^2,19,22–24^. Pufferfish nests possibly have similar roles in mating^15^. The simulation model could provide clues to correlate the nest pattern with the individual characteristics of pufferfish. We will examine this possibility in field experiments in the future study.

The simulation we made here was a simplified one, and only for the first half of nest construction. However, it is possible to improve on it by adding some conditions that could be obtained from the observation of the process of real nest formation. The accuracy of the excavation directionality increased from the early to middle stages. This is probably because the pufferfish tends to excavate along a ditch if the ditch is clear but neglects it if the ditch is unclear. It may be possible to confirm this by additional field experimentation.

Another interesting feature not included in the present simulation is the role of the outward excavation in the inner circle. Because the frequency of the outward excavation increased during nest construction (14/219 in early stage≪43/191 in middle stage), it must have a significant role. Fig. 1D suggests that the end points of the outward excavation were, in most cases, stopped just at the boundary between the inner circle and the outer ring region. We speculate the outward excavation functions to arrange the shape of the inner circle. This may also be confirmed by additional field experiments.

In conclusion, we found that the small pufferfish could construct a large and complex geometric structure by repeating rather simple behaviors. Although our simulation presented here remains as a simple and conceptional one, the parameters obtained from the video analysis can be used to further our understanding of the logic behind the complex nest construction by using more realistic simulations.

## Materials and Methods

### Video recording

After a pufferfish marked the central region on the sandy bottom on June 7, 2016, the videos were recorded on June 8 and 12, 2016 for the early- and middle-stage construction of the same nest, respectively, in Katetsu (28°08′N, 129°20′E), Amami-Oshima Island, Japan.

### Rectifying nest images

Assuming a nest can be approximated as a planar surface, we applied the projective transformation, or homography, to rectify a perspective image of the nest by removing the projective distortion. The coordinate of a pixel in the original nest image $({\boldsymbol{x}},{\boldsymbol{y}})$ was transformed to a new coordinate in its rectified image $({\boldsymbol{x}}\text{'},{\boldsymbol{y}}\text{'})$ by a homography. The transformation is expressed as $x\mbox{'}=\mathrm{Hx}$, where $x={[{\boldsymbol{x}}{\boldsymbol{y}}{\bf{1}}]}^{{\boldsymbol{t}}}$ and $x\mbox{'}={[{\boldsymbol{x}}\text{'}{\boldsymbol{y}}\text{'}{\bf{1}}]}^{{\boldsymbol{t}}}$ represent the coordinates of a pair of matching points, respectively. The $3\times 3$ homography matrix $H$ is calibrated with four point correspondences^25^. The transformation $H$ is then applied to the whole original nest image for the rectification. In this study, the four points in the rectified image were the corners. We developed a software where the corresponding pixels in the original nest image were manually selected by clicking them on the image. This method was applied to the video images to obtain photo images of pufferfish starting or ending excavations on the circular nest structures. The same homography matrix was used for the video images from the same observation date, during which the camera was set in the same position. 219 and 191 consecutive excavation images were obtained for the early- and middle-stage constructions, respectively.

### Estimation of the rectification

The method we used in the rectification is a standard one used in urban monitoring^26,27^. A homography transformation perspectively correctly warps a camera image as long as a captured scene is planar. Therefore, bumps in a captured nest cause bias on the location of a fish excavating the nest. We evaluated the bias by simulating the captured scene with the measured values of the camera’s intrinsic parameters (i.e., focal length, optical center, and lens distortion), the camera pose relative to the nest, and the maximum height variation of the nest from the bottom plane of the sea. We computed the bias for each point in the rectified image as follows. First, we projected the point to the bottom plane of the simulated sea. Second, we generated two points in the sea above and below the projected point, where the distance between each of the generated points and the projected point was the same as the maximum height variation. Third, we applied the homography transformation, which was the same as the one we used in rectifying the nest images, to these generated points to compute the positions in the rectified image. Finally, we computed the horizontal and vertical distances from each of these points to the original point. We repeated the above process for uniformly distributed 100 × 100 grid points in the rectified image. As a result, in the early-stage nest, the average of the horizontal distance was 0.8% (s.d. 0.5%) of the height (=width) of the rectified image, and that of the vertical distance was 6.8% (s.d. 0.6%). In the middle-stage nest, the average of the horizontal distance was 2.2% (s.d. 1.3%), and that of the vertical distance was 5.9% (s.d. 2.3%). The rectified videos of fish excavation of ditches are presented in the Supplementary Movies 4 and 5 that show the quality of the trace recording.

### Analysis of excavation trajectories

Each excavation trajectory was obtained by connecting the locations of the dorsal fin of the pufferfish every 0.5 second, on the rectified nest images. Each simplified trajectory (represented as a vector) was obtained by determining two positions on the sandy bottom on the rectified nest images: an entry position where the pufferfish initiates excavation, and an end position where the pufferfish stops excavation. The two positions were connected for each excavation as a vector towards the end position. Excavations with entry sites outside of the images were omitted from the analysis (0 and 9 excavations for the early and middle stages, respectively). The direction of the excavation was defined inward if the end position was closer to the center position than the entry position, and outward in the opposite case.

### Excavation steps in the simulation model

The entry positions, the direction of excavation, and the length of excavation were obtained, as described in the main text, with the measured parameter sets (Table 1), which were used after multiplying by a scaling factor S (S = 17.4 or 12.8 for the early- or middle-stage parameter sets, respectively). In each excavation, a model pufferfish excavates a defined length *m* from an entry position in the determined direction. At each time step *δ*_1_, heights were changed by excavation at cells on a line perpendicular to the direction of excavation, which has the length *b* + 2 *f* (*b*, the body width of pufferfish; and *f*, a factor of diffusion distance for each side) (Supplementary Fig. S4). With the averaging effect that decrease the amount of excavated sand, the total amount of excavated sand at each time step *T*′ was defined as follows.

$$T\text{'}=\{\begin{array}{ll}0,\, & \,\alpha  < -1\\ T(1+\alpha ), & -1\le \alpha \le 0\\ T, & \alpha  > 0\end{array}$$

Here, T is a defined amount of total excavated sand without the averaging effect, and *α* is the average heights at the excavation area (set as a rectangle). Averaged total sand *T*′ was excavated in *b*/*δ*_2_ cells (that may overlap) on the central *b* length of the line, and *T*′/2 sand was piled up in *f*/*δ*_2_ cells on the *f* length on either side, according to the standard normal distribution where 99% of values lie. These procedures were repeated *m*(1/*δ*_1_) times to complete one excavation step (that forms the rectangle region). *δ*_1_ = 0.25, *δ*_2_ = 0.25 and T = 0.2 for all the simulation experiments performed in this study.

## Electronic supplementary material

Supplementary information

Supplementary Movie 1

Supplementary Movie 2

Supplementary Movie 3

Supplementary Movie 4

Supplementary Movie 5

## Data Availability

The datasets generated and/or analyzed in the study are available from the corresponding authors on reasonable request.
