# Supplementary material for: Simple rules for construction of a geometric nest structure by pufferfish
Source: Sci Rep. 2018 Aug 17;8:12366. doi: 10.1038/s41598-018-30857-0 (PMC6098008; doi:10.1038/s41598-018-30857-0)
Supplement: Supplementary file 1 — Supplementary information [file 41598_2018_30857_MOESM1_ESM.docx]

**Supplementary Information**

Simple rules for construction of a geometric nest structure by pufferfish

Ryo Mizuuchi, Hiroshi Kawase, Hirofumi Shin, Daisuke Iwai, Shigeru Kondo


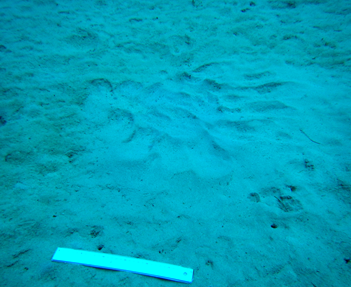


**Figure S1. The earliest stage of the nest construction.**

The photo image of the earliest stage (June 7, 2016) structure of the same nest examined in this study, reproduced from the previous study^20^.


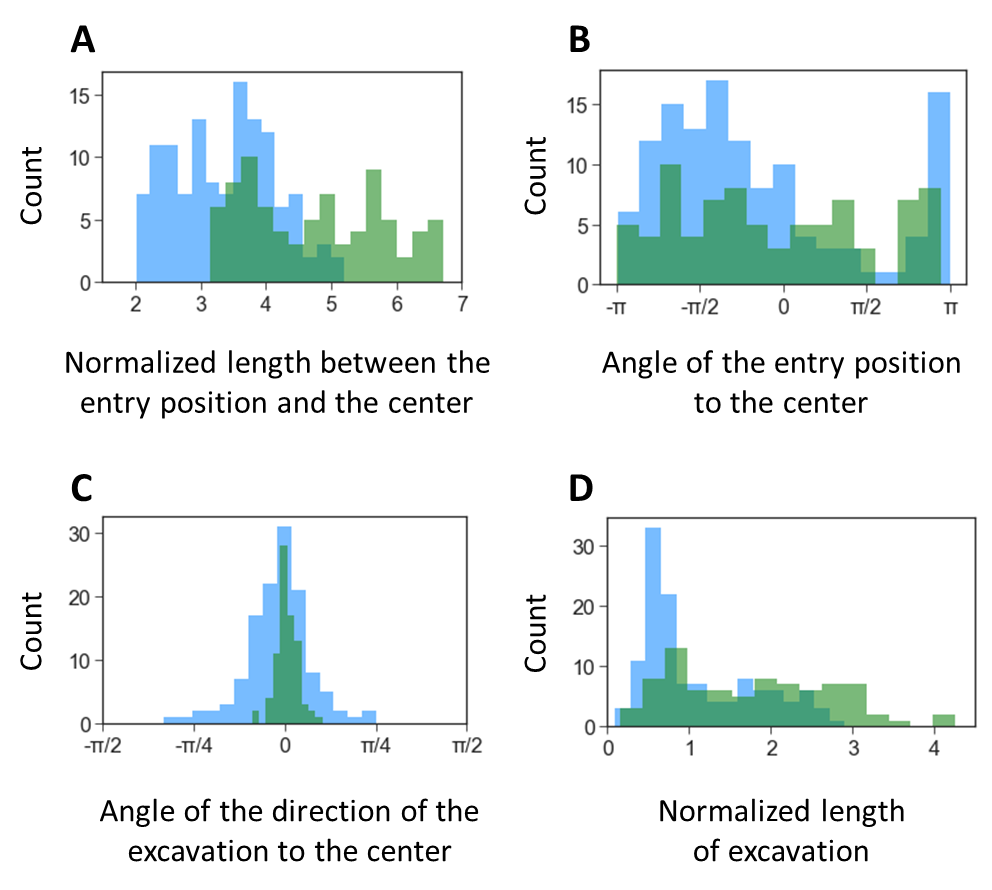


**Figure S2. Histograms for the measured values.**

(A) Length between the entry position and the center for all the analyzed excavations, normalized by the body length of the examined pufferfish. (B) Angle of the entry position to the center. (C) Angle of the direction of the excavation to the center, obtained as the angle between the vector of excavation and the vector toward the center position from the entry position. (D) Length of excavation, normalized by the body length of the examined pufferfish. For all the histograms, blue and green colors represent the measurement for the early- and middle-stage constructions, respectively.


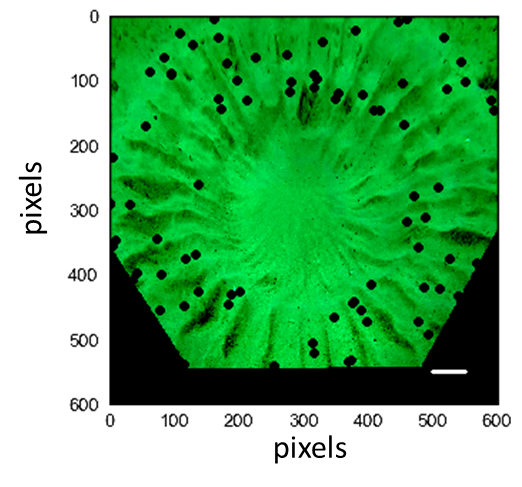


**Figure S3. Entry position during the middle-stage.**

For the middle-stage construction, the entry positions of the analyzed trajectories from the outer-ring region were plotted on the photo image of the nest (the same image as used in Fig. 1D). The contrast was adjusted to facilitate visualization.


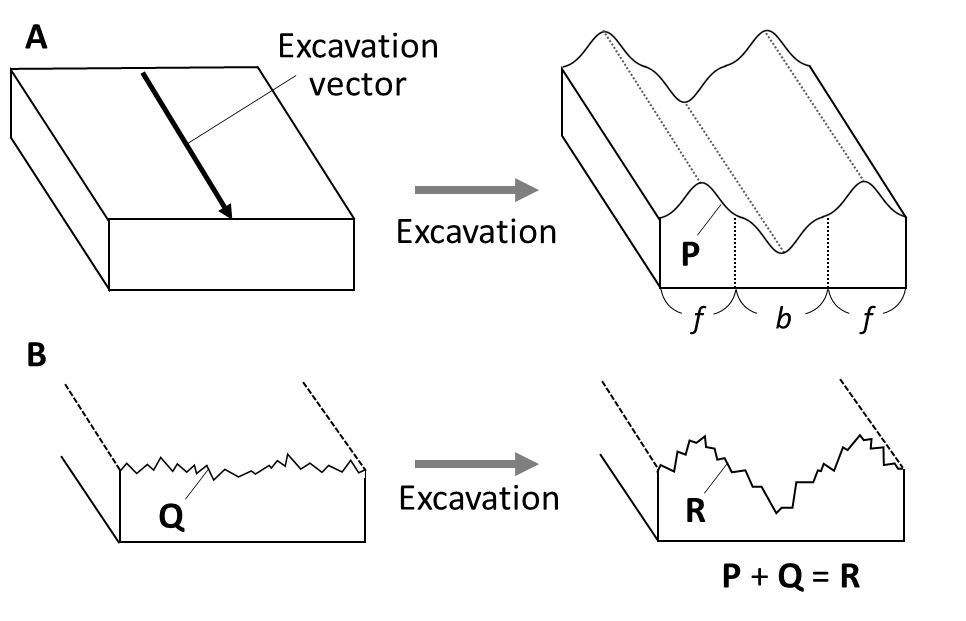


**Figure S4. Example of excavation on the simulation model.**

An example of excavation on a flat surface (A) or a ragged surface (B). The excavation occurs on a defined excavation vector, changing the heights on the rectangle area that is centered by the vector. By excavation, sand was excavated and piled up symmetrically on each side of the vector to form a normal distribution curve, depending on the body width of pufferfish *b* and the factor of diffusion distance *f.* P: The slope made from the flat initial condition. Q: the initial state with undulation. R: The slope made from the undulated initial condition.

**Table S1. Straightness index for trajectories in (Fig. 1B)**
